# 150. Improving Specificity of Congenital Cytomegalovirus Screening with Quantitative PCR on Saliva

**DOI:** 10.1093/ofid/ofaf695.052

**Published:** 2026-01-11

**Authors:** Anfal Marafie, Sarah Worley, Mary Kathryn Doud, Frank Esper, Hannah Wang

**Affiliations:** Cleveland Clinic Children's, Cleveland, OH; Cleveland Clinic, Cleveland, OH; Cleveland Clinic, Cleveland, OH; Cleveland Clinic Children's, Cleveland, OH; Cleveland Clinic, Cleveland, OH

## Abstract

**Background:**

Diagnosis of congenital cytomegalovirus (cCMV) is challenging. Screening is typically conducted on saliva followed by confirmatory testing on urine. Challenges in saliva screening include many false-positive (FP) results and relatively low throughput of currently FDA-approved platforms. Our institution validated CMV screening using quantitative PCR on the cobas 8800 (Roche) for infant saliva and urine. Following validation, we reviewed viral loads (VL) from a 2 year cohort to determine whether adjustment of reporting thresholds could improve the specificity of saliva screening.

**Figure:**
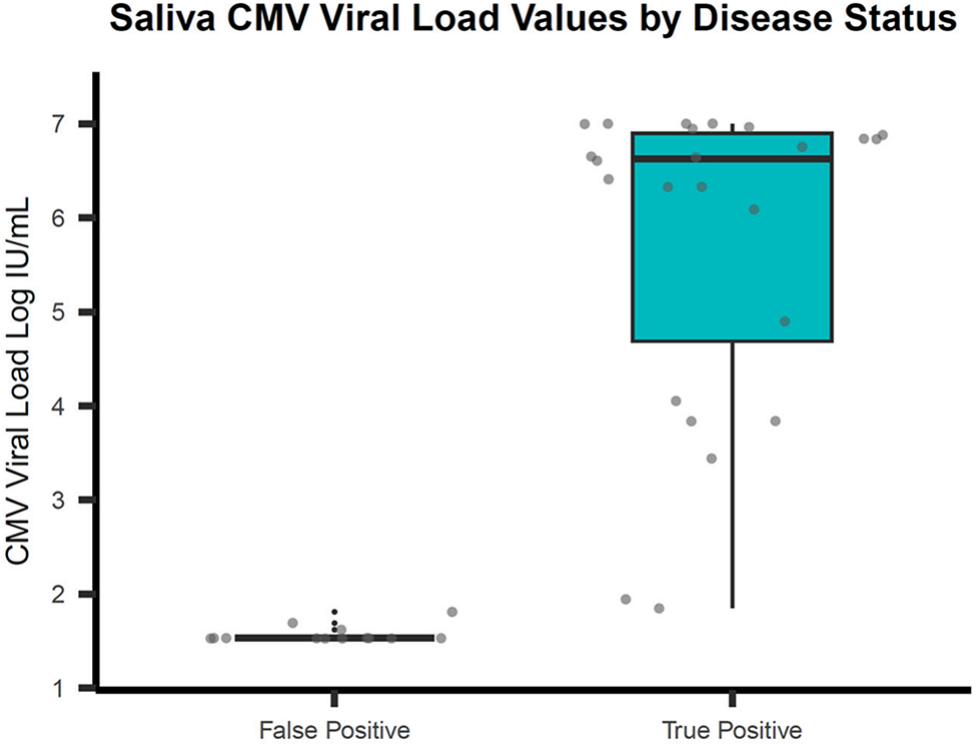
Figure 1 illustrates the distribution of log-transformed CMV DNA values in saliva stratified by disease status. Compared to the False Positive group, the True Positive group demonstrates a higher median CMV DNA level.

**Methods:**

From April 2023-March 2025, quantitative saliva and urine VL for infants < 21 days were tabulated. Saliva PCR sensitivity, specificity, positive and negative predictive value (PPV, NPV) were calculated at predefined VL thresholds and by Youden’s index. Urine PCR results were used to classify a saliva PCR result as a true vs. false positive (TP, FP). Specimens measuring above or below the limits of quantitation were assigned the maximal or minimal values of 7.01 log IU/mL or 1.53 log IU/mL respectively.

**Results:**

CMV PCR was positive in 37/3995 (0.93%) of saliva specimens obtained through targeted screening. Among saliva-positive, 24 (64.9%) had CMV detectable in urine (TP). Higher saliva VL significantly correlated with higher urine VL (Spearman correlation coefficient 0.88, 95% CI: 0.77-0.94). Saliva VL measured a median of 6.63 log IU/mL (IQR 4.69-6.90) for TPs, vs. 1.53 log IU/mL (IQR 1.53-1.53) for FPs (Figure 1). A total of 22 saliva negative infants also had subsequent urine testing; all were negative (TN).

Among 59 specimens with both saliva and urine PCR results, saliva PCR sensitivity was 100% (95% CI 85.8-100%), specificity was 62.9% (44.9-78.5%), observed PPV and NPV were 64.8% (54.5-74.0%) and 100% (84.6-100%). If saliva PCR positivity reporting threshold were adjusted such that those with CMV VL < 1.54 log IU/mL were reported as negative, specificity improved to 91.4% (76.9-98.2%) and observed PPV to 88.9% (73.1-95.9%), with no decrement in sensitivity or NPV.

**Conclusion:**

The quantitative CMV PCR performed in our lab is highly sensitive with excellent NPV in saliva swabs for cCMV screening. Assay specificity and PPV could be improved by adjusting the threshold of positivity to 1.54 log IU/mL.

**Disclosures:**

All Authors: No reported disclosures

